# Influence of capping agents on physicochemical properties and leukemic cytotoxicity of copper oxide nanoparticles biosynthesized using *Caesalpinia sappan* extract

**DOI:** 10.1371/journal.pone.0326791

**Published:** 2025-06-26

**Authors:** Mathurada Sasarom, Songyot Anuchapreeda, Wim E. Hennink, Siriporn Okonogi

**Affiliations:** 1 Department of Pharmaceutical Sciences, Faculty of Pharmacy, Chiang Mai University, Chiang Mai, Thailand; 2 Department of Medical Technology, Faculty of Associated Medical Sciences, Chiang Mai University, Chiang Mai, Thailand; 3 Center of Excellence in Pharmaceutical Nanotechnology, Chiang Mai University, Chiang Mai, Thailand; 4 Department of Pharmaceutics, Faculty of Science, Utrecht Institute for Pharmaceutical Sciences, Utrecht University, Utrecht, the Netherlands; Al-Azhar University, EGYPT

## Abstract

The aim of this study was to investigate the effects of capping agents on the physicochemical and biological properties, particularly their leukemic cytotoxicity, of copper oxide nanoparticles (CuONPs) using a *Caesalpinia sappan* extract as a reducing agent. Gelatin, polyethylene glycol 400 (PEG), polysorbate 80 (P80), octyl phenol ethoxylate, sodium lauryl ether sulfate and mannitol were added as capping agents to ensure colloidal stability of the formed CuONPs. As a control, CuONPs were also synthesized using gelatin and sodium borohydride as the capping and reducing agent, respectively. The physicochemical properties of the obtained CuONPs were determined using dynamic light scattering, zeta-potential measurements, energy dispersive X-ray spectroscopy, and Fourier-transform infrared spectroscopy. Their cytotoxic effects were investigated using normal human peripheral blood mononuclear cells (PBMC) and three strains of leukemic cell lines (KG1a, K562, and Molt4). The obtained CuONPs had a size range from 175–280 nm, with a reasonable size distribution between 0.2 and 0.4 and a negative zeta potential (range −30 to −35 mV) except the particles prepared using gelatin as a stabilizer which had a zeta potential of −3 mV. The CuONPs were incubated with both healthy PBMC and three types of leukemic cells to determine their IC_50_ values. The IC_50_ values of PEG-CuONPs and P80-CuONPs against healthy PBMC were 72.5 ± 5.8 and 85.0 ± 3.1 µg/mL, respectively, while that against the three strains of leukemic cells were in the range of 26–29 and 28–41 µg/mL, respectively. The results clearly demonstrate that the biosynthesized CuONPs using PEG and P80 as a capping agent exhibited the highest selectivity index defined as IC_50_ of the particles for PBMC/IC_50_ for leukemic cells. Therefore, these CuONPs are promising candidates for preclinical *in vivo* for leukemic treatments.

## Introduction

According to the American Cancer Society, cancer caused 18% of all deaths in USA and is the second leading cause of death in 2020 [1]. Leukemia is one of the most diagnosed malignant diseases and characterized by the uncontrollable generation of poorly differentiated white blood cells formed in the bone marrow. Different leukemias are known, such as myeloid leukemia, chronic myeloid leukemia, acute lymphoblastic leukemia, and multiple myeloma [2]. The success of the different treatment modalities of this type of cancer depends on several factors including type of leukemia, age of the patient, stage of the disease and patient history [3]. The current clinically used therapeutic modalities for leukemia are chemotherapy, radiation therapy and bone marrow transplantation. Compared to other modalities such as radiation therapy, donor lymphocyte infusions and stem cell transplantation, chemotherapy treatments are relatively simple. However, chemotherapeutic drugs have many unwanted and mostly toxic side effects on non-target healthy cells and organs. In addition, the development of multi-drug resistance of the treated cancer cells restricts therapeutic outcome. Liposomes [4–6] and polymer-based nanoparticles loaded with different types of anticancer agents (low molecular weight drugs as well as biotherapeutics such as siRNA) [7–9] are under investigation in preclinical as well as clinical studies. Some formulations are FDA approved to treat particularly solid tumors [10,11]. Nanomedicines are also used for the treatment of leukemia [12,13].

In the last decades, metal nanoparticles are under investigation for medical and pharmaceutical applications because of their attractive physical properties such as small size and as a consequence and large surface area, tailorable chemical composition, shape, and surface charge [14–17]. Importantly, they are also evaluated as anticancer imaging and therapeutic agents in both preclinical and clinical studies [18–20]. Among the many known metal nanoparticles, copper oxide nanoparticles (CuONPs) have gained attention due to their low cost, easy availability and possibilities to tailor their properties by the formulation and processing parameters. Importantly, they show beneficial antibacterial and anticancer effects that encourage their medical applications [21–23]. Copper is an essential nutrient and among others important for the catalytic activity of many enzymes [24]. However, at an overdose, copper shows genotoxic effects [25]. It is important to note that metallic Cu, Cu ions and Cu nanoparticles (CuONPs) show different toxicities [26–28]. The toxicity of CuONPs depends on their size and surface properties, shape, crystallinity and their colloidal stability [29,30]. CuONPs demonstrated anticancer activity against various cancer cell lines such as hepatocarcinoma, lung carcinoma, breast cancer, cervical carcinoma, and pancreatic cancer [31–34], and inhibited the proliferation of bladder cancer cells in a mouse model [35]. Regarding the biomedical applications of metal nanoparticles, Alizadeh et al. reported that CuONPs have broad potential for different biomedical applications including treatment of cancer [36]. The primary toxicity mechanism of CuONPs concerns the increased production of reactive oxygen species (ROS). However, further preclinical studies and subsequent clinical trials are necessary to demonstrate the therapeutic potential of these particles.

CuONPs are routinely prepared by adding under stirring a chemical reducing agent to an aqueous solution of a copper salt in which also a so-called capping agent is present, which covers the surface of the formed nanoparticles to ensure their colloidal stability [37,38]. As an alternative for chemical reducing agents, plant extracts are used to produce CuONPs. Plant-mediated synthesis has been considered an attractive route to prepare nanoparticles because it enhances their biocompatibility [39]. In our previous publications, CuONPs synthesized using *Caesalpinia sappan* extract as a reducing agent without a capping agent resulted in the formation of small particles (296 ± 7 nm) with high negative zeta potential (around −30 mV), and showed antioxidant, antiglycation and antimicrobial activities [40,41]. In the present study, we investigated the effect of capping agents on the particle physicochemical properties, their tolerability for healthy cells and the possible cytotoxic effect for leukemic cells of CuONPs synthesized by the same plant extract as a reducing agent. CuONPs synthesized using sodium borohydride and gelatin as reducing and capping agents, respectively, were used as a control. This study investigates whether CuONPs, synthesized using *C. sappan* extract, are cytotoxic for leukemic cells (K562, KG1a, and Molt4).

## Materials and methods

### Materials

Copper sulfate pentahydrate, sodium borohydride, and potassium bromide for Fourier-transform infrared spectroscopy were from Merck (Darmstadt, Germany). Polyethylene glycol 400 (PEG), polysorbate 80 (P80), and Histopaque-1077 were from Sigma (MO, USA). Octyl phenol ethoxylate (Tx) was from Loba Chemie (Maharashtra, India). Sodium lauryl ether sulfate (SLES), mannitol (Man) and gelatin type A (G) were from Ajax Finechem Pty ltd (New South Wales, Australia). Fetal bovine serum (FBS), penicillin, streptomycin, L-glutamic acid, Iscove’s modified Dulbecco’s medium (IMDM), and Roswell Park Memorial Institute medium (RPMI 1640) were purchased from GIBCO Invitrogen TM (Waltham, MA, USA). 3-(4,5-Dimethylthiazol-2-yl)-2,5-diphenyltetrazolium bromide (MTT) reagent was obtained from BioVision (Milpitas, CA, USA). Doxorubicin hydrochloride injection IP 50 mg/25 mL was from Fresenius Kabi (Bangkok, Thailand). Other chemicals and solvents were of analytical grade.

### Preparation of *C. sappan* extract

*C. sappan* was identified and collected by a botanist of the botanical herbarium of the Faculty of Pharmacy, Chiang Mai University, Thailand, to obtain the reference voucher specimen (no. 002276). The preparation of plant extract was carried out according to our previously published method [40]. Briefly, five grams of heartwood powder of *C. sappan* was mixed with 50 mL of water. The resulting mixture was stirred at 500 rpm overnight and then filtered through Whatman’s No.1 filter paper. The filtrate was centrifuged at 3000 × *g* for 10 min to eliminate big particles. Subsequently, the filtrate was lyophilized using a Beta 2–8 LD-plus freeze dryer (Martin Christ, Osterode am Harz, Germany). The obtained extract was analyzed for its reducing activity using the ferric reducing antioxidant power (FRAP) as described in a previously described method [42]. Briefly, the FRAP reagent was freshly prepared by mixing 1 mL of 10 mM TPTZ solution in 40 mM HCl with 1 mL of 20 mM FeCl_3_ and 10 mL of 0.3 M acetate buffer pH 3.6. Next, 20 µL water with the aqueous extract (10 mg/mL) was mixed with 180 µL of FRAP reagent in a 96 well plate. After 5 min, the absorbance of the reaction mixture was recorded at 595 nm using a microtiter plate reader (Spectrostar Nano, BMG Labtech, Ortenberg, Germany). The reducing power activity of the extract was evaluated by calculating the amount of Fe2+ produced by the aqueous extract using FeSO_4_ for calibration.

### Biosynthesis of CuONPs with and without capping agents

CuONPs were prepared by reducing copper sulfate dissolved in water with the aqueous extract of *C. sappan* according to our previously described method [40]. Briefly, 6 different solutions of 19 mL of 10 mM copper sulfate (or 44 mg per solution) were heated to 70°C for 10 min under magnetic stirring. These solutions were subsequently added to 10 mL water or 10 mL of water with 10 mg/mL G, PEG, P80, Tx, SLES, or Man. Next, 60 mg of the obtained lyophilized powder of the extract of *C. sappan* was dissolved in 6 mL water. After 5 min of sonication, 1 mL of the extract was added to the different copper sulfate solutions which were subsequently stirred for 30 min at 70°C. Next, the acid mixtures (pH ~ 3) were adjusted to pH 10 by addition of 400 µL of a 1 M NaOH solution and stirred for 2 h at 70°C. The excess extract was removed by centrifugation of the nanoparticle dispersion at 8000 × *g* for 30 min. The obtained pellet was redispersed in 30 mL milli-Q water, and this precipitation/washing procedure was repeated two times, after which the precipitate was dispersed in absolute ethanol and dried at 60°C for 8 h. The obtained CuONPs were collected and named CuONPs (uncapped NPs), GCS-CuONPs, PEG-CuONPs, P80-CuONPs, Tx-CuONPs, SLES-CuONPs, and Man-CuONPs. The particles were transferred into light-protecting containers and stored in a desiccator at room temperature until use.

### Synthesis of CuONPs prepared without using *C. sappan* extract

The preparation of CuONPs without using *C. sappan* extract was carried out using sodium borohydride as a reducing agent and gelatin as a capping agent according to the method previously described with some slight modifications [43]. Briefly, 10 mL of 0.2 M copper sulfate (or 5.0 g per solution, a higher concentration than in the section of biosynthesis of CuONPs with and without capping agents) and 10 mL of 1 mg/mL gelatin also dissolved in water were mixed and heated at 70°C for 30 min under magnetic stirring. Then, 10 mL of 0.4 M sodium borohydride (or 0.15 g per solution) was slowly dropped in about 5–10 min into the solution. The pH of this mixture was adjusted from approximately 2–12 by 6 mL of 1 M sodium hydroxide, then heated at 70°C with magnetic stirring for 30 min. After that, the obtained CuONPs, further referred to as G-CuONPs, were isolated as described in the section of biosynthesis of CuONPs with and without capping agents.

#### Particle characterization.

In the present study, the synthesized CuONPs were characterized using a Malvern Zetasizer Nano-ZS (Malvern Instruments, Worcestershire, UK). This instrument measures particle size and size distribution using dynamic light scattering (DLS), which analyzes fluctuations in scattered light intensity caused by the Brownian motion of the particles [44]. In addition, using this machine, the zeta potential of the particles was also determined by electrophoresis and laser doppler velocimetry. Five hundred microliters of 0.2 mg/mL of the different CuONPs in Milli-Q water were diluted with 500 μL of 20 mM HEPES pH 7.4. The dispersions were sonicated for 30 min before measurement. The size measurements were taken at a fixed angle of 173°. The particle size distribution is expressed as the polydispersity index (PDI). The zeta potential of the CuONPs (dispersed in the same medium as used for DLS measurements) was determined using a Zetasizer (Malvern, Worcestershire, UK) and calculated by applying the Smoluchowski model [45]. The measurements were performed in triplicate.

#### Morphology and chemical composition of the CuONPs.

The morphology of the synthesized CuONPs was examined using a transmission electron microscope (TEM) (JEM2010 JEOL, Tokyo, Japan) at an acceleration voltage at 200 kV. The samples were prepared as follows: 500 μL of 0.2 mg/mL of the selected CuONPs in Milli-Q water was diluted with 500 μL of 20 mM HEPES pH 7.4. The dispersions were sonicated for 30 min before the measurements. Then, 20 µL of the samples were dropped on discharged 200 mesh copper grids coated with formvar and carbon (FCF-200 mesh Cu, Electron Microscopy Sciences, Hatfield, PA, USA). The excess solution was removed by filter paper and left for air-drying at room temperature for one day before measurement.

Energy dispersive X-ray (EDX) was selected for chemical composition analysis of the synthesized CuONPs. This technique provides an overall mapping of the sample by analyzing near-surface elements and estimating the elemental proportions at different positions. In general, EDX is used in conjunction with SEM. An electron beam with an energy of 10–20 keV strikes the surface of the conducting sample, causing X-rays to emit from the material, with the energy of the emitted X-rays depending on the material under examination. In the present study, EDX (JEM-2100VL, JEOL, Tokyo, Japan) was used to analyze the presence of copper, carbon, and oxygen.

#### Molecular interaction analysis of the CuONPs.

Fourier transform infrared (FTIR) was used for the identification of both organic and inorganic materials and possible molecular interactions. The presence of the capping agents in the CuONPs was investigated using an FTIR spectrometer (Thermo Nicolet/470FT-IR spectrometer, Nicolet Nexus, Madison, USA) with a resolution of 4 cm^–1^. Ten mg of dried powders of each sample were mixed with around 500 mg KBr and then analyzed in the range of 450–4000 cm^–1^.

### Ethics statement

Peripheral blood mononuclear cells (PBMC) used in this study were from volunteers who had signed an informed written consent form approved by Human Research Ethics Unit of the Faculty of Associated Medical Sciences, Chiang Mai University (AMSEC-66EM-100). The recruitment period for this study was from January 8 to February 29, 2024.

### Isolation of normal cells and culture conditions

The PBMC were obtained from 5 healthy donors (both female and male, aged 18–36 years). The criteria for donor selection were that the donors had a healthy lifestyle meaning that they were not overweight, smokers, or alcohol consumers, and were free from the following diseases: diabetes, hypertension, heart disease, cerebrovascular disease, cancer, or microbial infections. Blood samples were collected after the donors agreed to and signed a consent form. The blood was collected by venipuncture and transferred into heparin coated tubes [45]. The collected blood was diluted with the same volume of PBS (1.37 M sodium chloride, 27 mM potassium chloride, 101.4 mM phosphate buffer, 17.6 potassium phosphate monobasic, pH 7.4). Histopaque-1077 was added to the diluted blood at a volume ratio of 3:1 and centrifuged at 1000 × *g* for 30 min to isolate PBMC from erythrocytes and granulocytes. The PBMC layer was collected and washed two times with PBS and resuspended in RPMI-1640 medium to assess cell survival rate after incubation with the different CuONPs using the MTT assay (details are provided in the section of MTT assay).

### Leukemic cell lines and culture conditions

Three strains of leukemic cell lines, namely KG1a (leukemia stem cells), K562 (chronic myelogenous leukemia) and Molt4 (acute lymphoblastic leukemia) were used in this study. KG-1a was purchased from ATCC (Manassas, VA, USA). K562 cells and Molt4 cells were purchased from RIKEN BRC Cell Bank (Ibaraki, Japan). Doxorubicin was from Fresenius Kabi Ltd (Bangkok, Thailand). The KG1a cells were cultured in IMDM supplemented with 200 mg/mL FBS, 100 units/mL penicillin and 100 μg/mL streptomycin. The K562 and Molt4 cells were cultured in RPMI-1640 containing 100 mg/mL fetal calf serum, 1 mM L-glutamine, 100 units/mL penicillin and 100 μg/mL streptomycin. The cell lines were maintained in a humidified incubator at an atmosphere of 95% air and 5% CO_2_ at 37°C. After incubation of the cells with the different CuONPs, their survival rate was assessed utilizing the MTT assay (details are provided in the following section).

### MTT assay

MTT assay was performed to investigate the cytotoxicity of the different CuONPs against normal cells and leukemic cell lines [46,47]. In short, 100 μL of cell suspensions at the following cell concentrations, 2.5 × 10^5^ cells/mL for PBMC, 1.5 × 10^4^ cells/mL for KG1a, and 1.0 × 10^4^ cells/mL for K562 or Molt4 were pipetted into the well of the flat-bottomed 96-well plates. Then, 100 μL of the different CuONPs at concentrations ranging from 3.125 to 200 μg/mL and doxorubicin (concentrations ranging from 0.01 to 4 μg/mL) as a positive control were added to cells. A medium without CuONPs was used as a negative control. The cells were further incubated for 48 h at 37°C. Subsequently, 15 μL of 0.2 mg/mL MTT dye solution was pipetted into each well and the plate was further incubated for 4 h at 37°C. The MTT dye is converted by NAD(P)H-dependent cellular oxidoreductase from viable mitochondria into insoluble formazan crystals [48]. After the supernatants of the well were removed, the formazan crystals were dissolved in 200 μL of DMSO, and the absorbance of the solutions was measured at 578 nm on a microplate reader (Metertech, Taipei, Taiwan), using 630 nm as a reference wavelength. The percentage of viable cells was calculated by the following equation: Cell viability (%) = (*OD*_*t*_/*OD*_*c*_) × 100%, where *OD*_*t*_ is the average optical density of the test sample and *OD*_*c*_ is the average optical density of the negative control. The 50% inhibitory concentration (IC_50_), defined as the concentration that inhibited cell growth by 50% compared to the negative control, was determined by plotting the %cell viability against the concentration of CuONPs. The selectivity index (SI) was determined to assess the selective cytotoxicity of samples to cell lines tested. The index was calculated using the formula: SI = IC_50_ of PBMC cells/ IC_50_ tested leukemic cell lines.

### Statistical analysis

The experiments were performed in triplicate. The results are expressed as mean ± standard deviation (SD) or mean ± standard error of the mean (SEM). Statistical analysis was performed on SPSS software version 20 for Windows. Differences between groups were determined by one-way analysis of variance (ANOVA) followed by Ducan’s post hoc test. A *p*-value<0.05 is considered statistically significant.

## Results and discussion

### Preparation and characteristics of the CuONPs

In the current study, CuONPs were prepared using an established method of reduction of copper sulfate dissolved in water using reducing agents. This results in the formation of colloidal particles composed of a mixture of copper (Cu), copper (II) oxide (CuO), and copper (I) oxide (Cu_2_O) [49,50]. As pointed out by Guzman *et al*. [50], the formation of CuONPs is a complex process and during the formation many reactions occur in series or parallel. In the simplest form, a reducing agent donates electrons to reduce Cu^2+^ to yield metallic copper and copper oxides. These compounds are insoluble in water, and therefore, the formed copper particles aggregate. Importantly, when capping agents are present in the reaction mixture the aggregation rate is controlled, and copper oxide nanoparticles are formed. The capping agents are adsorbed onto the surface of the formed particles, and thereby stabilize them by providing steric and/or electrostatic repulsion and thus preventing further particle growth [50,51]. In this study, an extract of *C. sappan*, exhibiting a reducing capacity of 57.8 ± 0.1 mM Fe^2+^/10 mg of extract, was used as a reducing agent. The characteristics of the resulting nanoparticles (NPs) were then compared to those formed without *C. sappan*, where sodium borohydride was used as the chemical reducing agent. The yield and characteristics of the different NPs using a Malvern Zetasizer Nano-ZS are summarized in Table 1. This table shows that CuONPs were obtained in a good yield (57–92%). The particle size of CuONPs prepared without a capping agent and the plant extract as a reducing agent was 428 ± 8 nm. When capping agents were present in the reaction mixtures, the obtained particles (except one formulation, further discussed below) were substantially smaller with diameters ranging from 176 ± 1–284 ± 10 nm.

**Table 1:** Yield and particle characteristics of the synthesized CuONPs.

Capping agent	Sample	Yield and particle characteristics***			
		Yield (%)	Particle size (nm)	PDI	Zeta potential (mV)
–	CuONPs*	75.1 ± 7.7^bc^	428 ± 8^a^	0.297 ± 0.026^a^	–42.5 ± 0.3^a^
Gelatin	GCS-CuONPs*	74.8 ± 4.7^bc^	>1000^b^	0.700 ± 0.078^b^	–1.8 ± 0.6^b^
PEG400	PEG-CuONPs*	96.2 ± 20.3^a^	176 ± 1^a^	0.173 ± 0.012^a^	–34.5 ± 1.3^a^
Polysorbate80	P80-CuONPs*	78.7 ± 14.5^b^	200 ± 7^a^	0.382 ± 0.035^ab^	–30.2 ± 0.7^a^
Octyl phenol ethoxylate	Tx-CuONPs*	76.0 ± 8.0^bc^	242 ± 3^a^	0.321 ± 0.007^ab^	–32.9 ± 2.9^a^
Sodium lauryl ether sulfate	SLES-CuONPs*	91.8 ± 1.3^ab^	235 ± 9^a^	0.272 ± 0.044^a^	–38.2 ± 0.9^a^
Mannitol	Man-CuONPs*	81.8 ± 1.1^b^	205 ± 2^a^	0.234 ± 0.018^a^	–34.5 ± 1.1^a^
Gelatin	G-CuONPs**	57.8 ± 2.3^c^	284 ± 10^a^	0.364 ± 0.011^ab^	–2.7 ± 0.2^b^

* CuONPs prepared with *C. sappan* extract, ** prepared without *C. sappan* extract, *** results are expressed as the mean and standard deviation of three independently prepared batches. Lowercase letters indicate significant diffences (*p* < 0.05).

The results showed that the particles had an acceptable size distribution, as reflected by their PDI of around 0.3. The particles in 10 mM HEPES had a negative zeta potential in the range from −1.8 ± 0.6 to −42.5 ± 0.3 mV. The use of the plant extract in combination with gelatin did not result in the formation of NPs but rather in larger aggregates (~3.5 µm). The zeta potential of the synthesized CuONPs was negative (−1.8 ± 0.6 to −42.5 ± 0.3 mV) due to the adsorption of hydroxide ions onto the surface of the particles [52–55], which results in electrostatic repulsion of the particles. The capping agents obviously shield the surface charge resulting in a slight reduction of the zeta potential from −42 mV for the particles prepared without a capping agent to around on the average −35 mV for the particles with the different capping agents. As an exception, the NPs stabilized with gelatin had a close to zero zeta potential. The used gelatin is a mixture of proteins that has isoelectric points ranging from pH 5–10. Consequently, at pH 7.4 of the HEPES buffer in which the particles were dispersed, the overall charge of the surface adsorbed proteins is close to neutral explaining, in combination with the tight packing of the proteins on the surface, the almost neutral zeta-potential [56,57].

### Morphology and chemical compositions of the synthesized CuONPs

**Fig 1:**
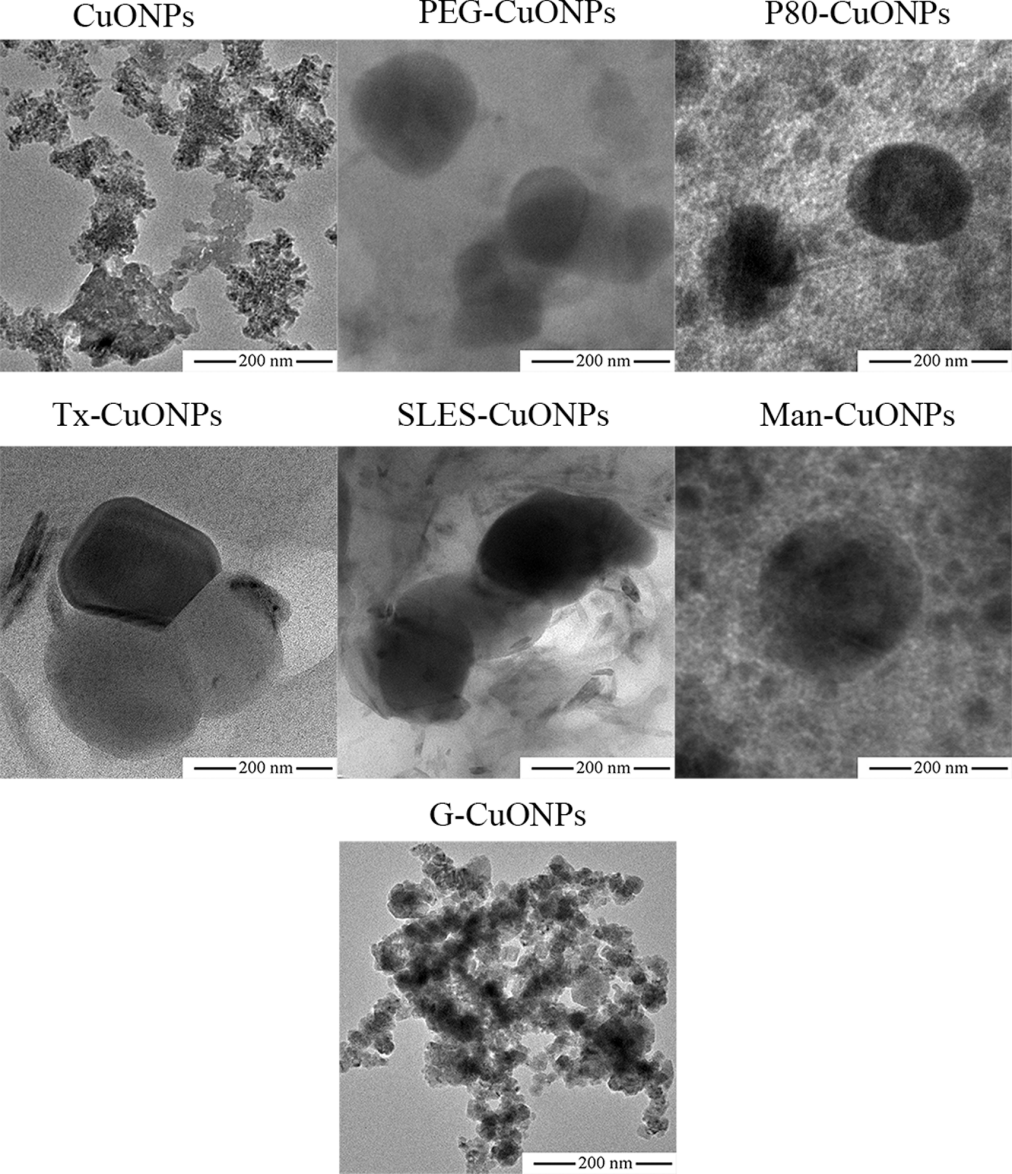
TEM images of the synthesized CuONPs.

**Fig 2:**
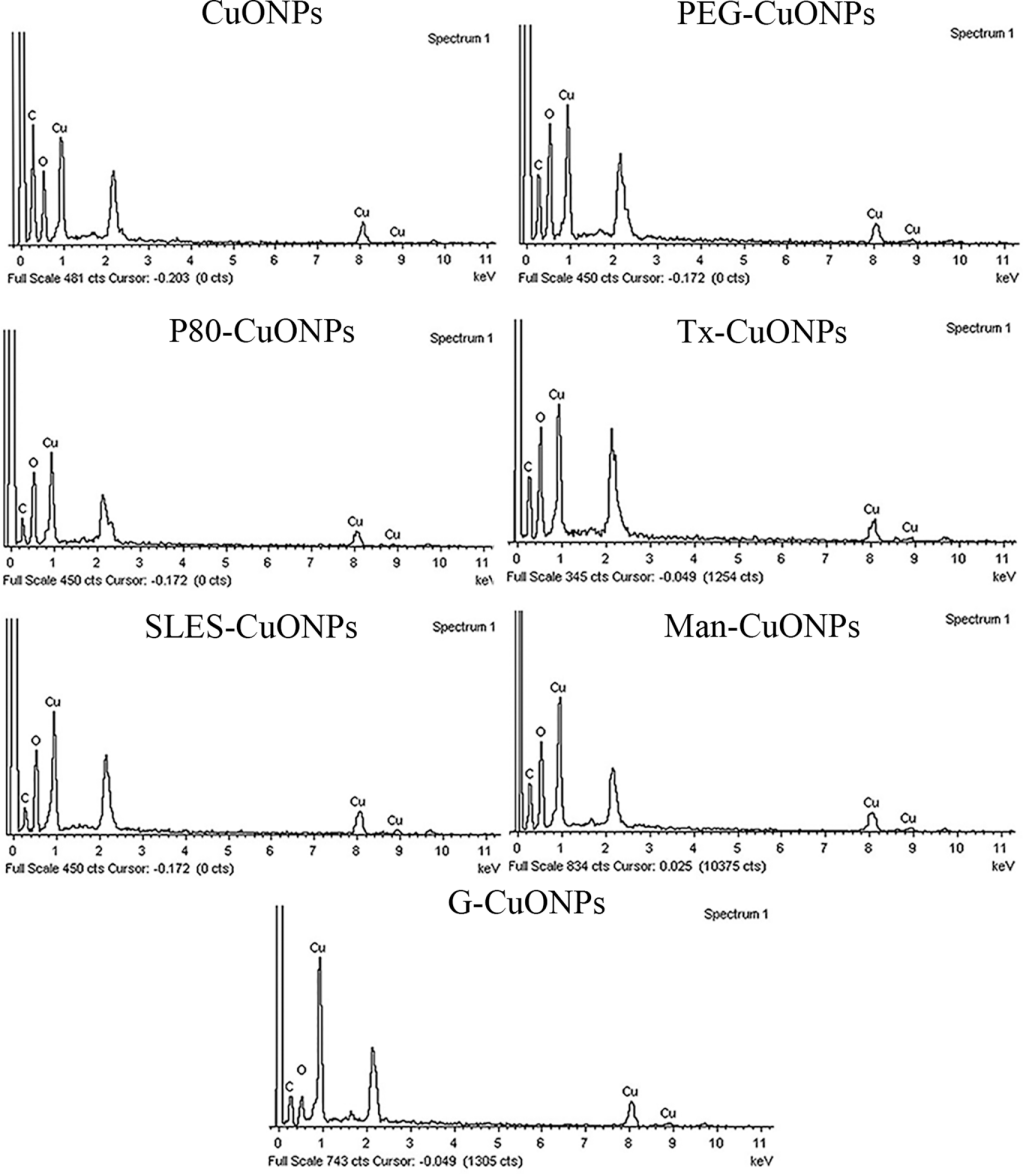
EDX spectra of the synthesized CuONPs.

As shown in Fig 1, CuONPs prepared without a capping agent strongly agglomerated. In contrast, less agglomeration was observed for the biosynthesized CuONPs, except when gelation was employed as a capping agent. The particles size of the obtained CuONPs as observed with TEM was in correspondence with that characterized using DLS. The EDX patterns of the different CuONPs demonstrated, as expected, the presence of copper, oxygen, and carbon, as shown in Fig 2. No substantial differences in compositions of the different CuONPs were observed.

### FTIR analysis of the CuONPs

**Fig 3:**
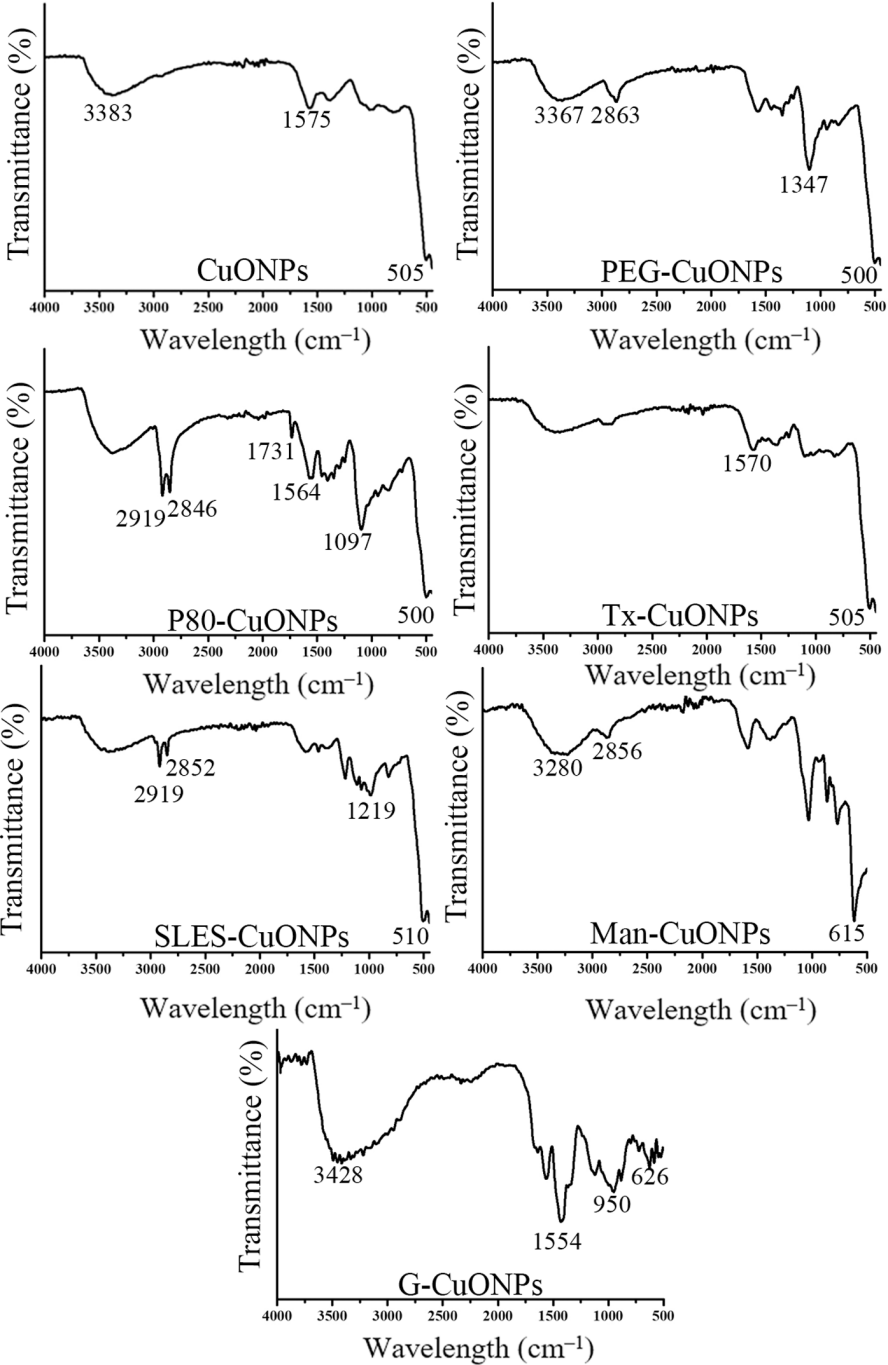
FTIR spectra of the synthesized CuONPs.

**Fig 4:**
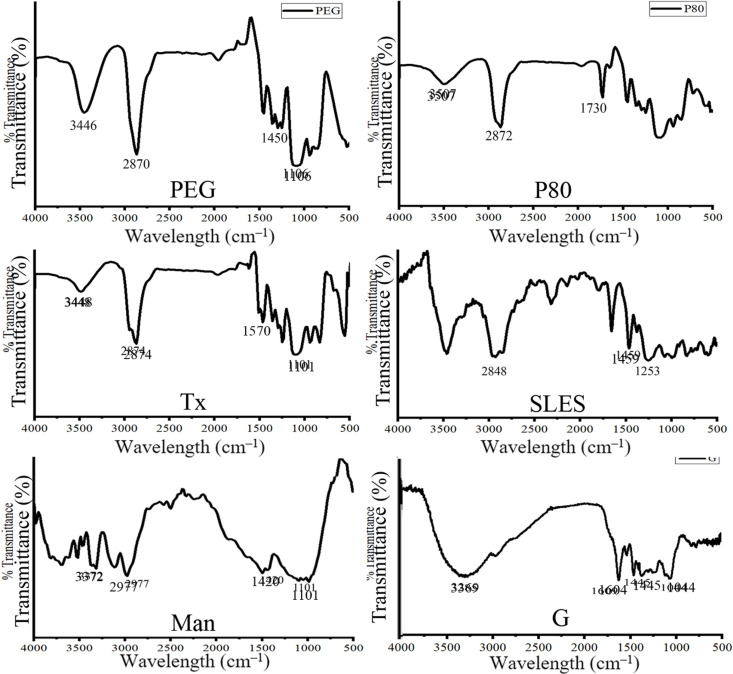
FTIR spectra of capping agents.

FTIR analysis of the CuONPs was investigated to establish the presence of the capping agents in the obtained NPs. The results are shown in Fig 3. The analysis of different capping agents was also performed as a control, and the results are reported in Fig 4. The FTIR results of the different CuONPs demonstrate the presence of peaks between 500–700 cm^–1^, which are assigned to the stretching vibrations of copper oxide and copper hydroxide [58–60]. In the FTIR spectrum of CuONPs synthesized using the plant extract as a reducing agent, a broad peak around 3383 cm^–1^ assigned to hydroxyl groups is presented. A peak at 1575 cm^–1^ is observed which likely can be assigned to C = O stretching of the carboxylic groups present in compounds of the extract. In the FTIR spectrum of PEG-CuONPs, the peaks at 3367, 2863, and 1347 cm^–1^, which are assigned to the stretching and bending vibration bands of O-H, C-H, and C-O groups, respectively, present in PEG. The spectrum of P80-CuONPs shows two peaks at 2919 and 2846, 1731, 1564, and 1097 cm–1 are assigned to asymmetric and symmetric vibrations of CH_2_, C = O bond, vibrations of C-H, and C-O stretching, respectively, present in polysorbate 80. In the spectrum of Tx-CuONPs, a peak at 1570 cm^–1^ is assigned to C-H bending present in octyl phenol ethoxylate. In the spectrum of SLES-CuONPs, peaks at 2919, 2852, and 1219 cm^–1^ indicate asymmetric and symmetric vibrations of CH_2_ and SO_2_ stretching vibration, respectively. The spectrum of Man-CuONPs shows a broad peak at 3280 cm^–1^, which is assigned to OH stretching vibration, and a peak at 2856 cm^–1^ for C-H stretching vibration. For the FTIR spectrum of G-CuONPs, a broad absorption at around 3428 cm^–1^ and a peak at 1554 cm^–1^ indicate OH and C-N stretching vibration and indicate the presence of gelatin. The absorption bands below 1000 cm^–1^ are detected in all synthesized CuONPs and are assigned to the metal oxide [61]. In conclusion, FTIR analysis demonstrates that capping agents are present in the CuONPs, and most likely at the surface of the nanoparticles thereby contributing to their colloidal stability.

### Cytotoxicity analysis

The cytotoxicity of copper sulfate, *C. sappan* extract, the different CuONPs, and doxorubicin, as a positive control, against leukemic cells and normal cells was investigated, and the results are shown in Table 2. The results show that the positive control, doxorubicin, is rather toxic for the leukemic cell lines with IC_50_ values from 0.4 ± 0.0 to 0.7 ± 0.1 µg/mL. On the other, the PBMC were much less sensitive for this drug (IC_50_ was 1.8 ± 0.0 µg/mL). This means that the specific cytotoxicity or selectivity index of doxorubicin (defined as IC_50_ PBMC/IC_50_ cancer cells) is 2.6–4.5 and confirms the validity of the selected cell lines to study the selectivity index of the different NPs.

**Table 2:** IC_50_ and SI values of *C. sappan* extract, copper sulfate, and the synthesized CuONPs against PBMC and the leukemic cell lines.

Samples*	PBMC	KG1a		K562		Molt4	
	IC_50_ (μg/mL)	IC_50_ (μg/mL)	SI	IC_50_ (μg/mL)	SI	IC_50_ (μg/mL)	SI
*C. sappan* extract	66.7 ± 4.0^c,d,e^	20.9 ± 3.7^b^	3.2 ± 1.0^a^	>100.0^e^		>100.0^e^	
Copper sulfate	73.9 ± 1.8^d,e,f^	>100.0^e^		>100.0^e^		>100.0^e^	
CuONPs	54.1 ± 6.2^c^	79.5 ± 9.0^d^	0.7 ± 0.2^c^	73.2 ± 4.2^d^	0.7 ± 0.0^d^	90.8 ± 7.2^d^	0.6 ± 0.0^c^
PEG-CuONPs	72.5 ± 5.8^c,d,e,f^	29.3 ± 6.2^b^	2.5 ± 0.3^b^	26.3 ± 4.1^b^	2.8 ± 0.3^b^	29.2 ± 3.8^b^	2.5 ± 0.3^b^
P80-CuONPs	85.0 ± 3.1^e,f^	28.2 ± 1.3^b^	3.0 ± 0.1^a,b^	35.7 ± 6.1^b^	2.4 ± 0.4^b^	41.9 ± 3.2^b^	2.0 ± 0.2^b^
Tx-CuONPs	27.3 ± 2.3^b^	57.1 ± 0.8^c^	0.5 ± 0.1^c,d^	25.5 ± 2.5^b^	1.1 ± 0.1^d^	90.5 ± 5.4^d^	0.3 ± 0.0^d^
SLES-CuONPs	36.8 ± 2.9^b^	35.6 ± 3.4^b^	1.0 ± 0.1^c^	30.5 ± 2.6^b^	1.2 ± 0.1^d^	73.7 ± 4.7^c^	0.5 ± 0.0^c,d^
Man-CuONPs	87.4 ± 3.5^f^	>100.0^e^		54.7 ± 7.1^c^	1.6 ± 0.2^c^	>100.0^e^	
G-CuONPs	62.6 ± 4.0^c,d,e^	>100.0^e^		83.9 ± 0.1^d^	0.7 ± 0.0^d^	>100.0^e^	
Doxorubicin	1.8 ± 0.0^a^	0.7 ± 0.1^a^	2.6 ± 0.3^b^	0.5 ± 0.1^a^	3.6 ± 0.4^a^	0.4 ± 0.0^a^	4.5 ± 0.1^a^

* Results of IC_50_ values of the samples against the leukemic cell lines are expressed as mean±SD (n = 3) and that against PBMC are expressed as mean±SEM (n = 5). Lowercase letters indicate significantly different (*p* < 0.05).

The results also show that the *C. sappan* extract was rather cytotoxic for KG1a cells with an IC_50_ value of 20.9 ± 3.7 µg/mL, but the extract had no toxicity towards K562 and Molt4 cells (IC_50_ > 100.0 µg/mL). In addition, the extract showed some toxicity towards PBMC (IC_50_ was 66.7 ± 4.0 µg/mL). *C. sappan* has shown to have anticancer activity against some cancer cells such as HeLa, KG1 and KG1a cells through a variety of mechanisms, including cell cycle arrest, induction of apoptosis pathway, inhibition of protein phosphorylation, and inhibition of nuclear factor kappa light chain enhancer of activated B cells (NF-kB) and matrix metallopeptidase 2 (MMP-2) [62–64]. The mechanisms regarding the cytotoxicity of CuONPs were reviewed in several studies [28,47]. In short, CuONPs exert toxicity to cells through various mechanisms, including the induction of formation of reactive oxygen species (ROS), damage to the cell membranes, a decrease in ATP production, and induction of apoptosis or autophagy.

Copper sulfate did not show cytotoxic effects toward the different leukemic cell lines-even at the highest concentration tested (100 μg/mL) whereas the PBMC showed some sensitivity towards this agent (IC_50_ was 67 μg/mL). The observed toxicity of copper sulfate against the different cancer cells is in agreement with a publication of Chen *et al*. who found that the IC_50_ of this salt for HeLa cells was 56 μg/mL [65]. The cytotoxicity towards cancer cells results from oxidative stress induced by the generation of ROS. ROS formation triggered by chemotherapeutic agents, results in cell death through various pathways, including apoptosis, autophagy, and necrosis. Elevated ROS concentrations result in structural damage of essential cellular constituents, such as DNA, proteins, and lipids, ultimately leading to cellular dysfunction and finally to the death of cancer cells [66]. Generally, copper is an essential micronutrient for physiological functions in the body and plays a role in the redox activity in enzyme-catalyzed reactions [24,67]. When copper is present in excessive concentrations in cells, it can catalyze the production of ROS, leading to the damage of lipids, proteins and DNA. In various types of tumor cells, high concentrations of copper showed to promote tumor growth and development [68]. Moreover, copper accumulation in cancer cells can induce the production of ROS in cells [69]. Our present results agree with previous findings that copper nanoparticles are substantially more cytotoxic than soluble copper salt [70,71]. Likely soluble copper ions do not pass cellular membranes, whereas previous studies demonstrated that CuONPs are taken up by cells through endocytosis and subsequently release copper ions in cells [72–74]. This in turn results in copper-induced ROS formation, glutathione depletion, and oxidative DNA lesions finally resulting in cell death. Besides, the solubilization of copper ions from internalized nanoparticles affects biological systems [75]. Uncoated metal nanoparticles often tend to aggregate. Importantly, capping agents can prevent aggregation and, therefore, play a crucial role in controlling particle size and preventing instability. Additionally, they influence the biological activities and toxicity of metal nanoparticles [76].

The IC_50_ values of the different capping agents against leukemic cells were also determined as control and reported in Table 3. The results suggest that the possible presence of the capping agents in the formulations likely does not contribute to the observed cytotoxic effect since their IC_50_ values, except for surfactant triton X, were above 100 μg/ml. Interestingly, PEG-CuONPs and P80-CuONPs showed low toxicity to the PBMC with IC_50_ of 72.5 ± 5.8 and 85.0 ± 3.1 µg/mL, respectively. It has been shown in other studies that PEGylation of zinc [77], selenium [54], and iron oxide [78] nanoparticles enhanced their anticancer activity. Importantly, the results show that PEG-CuONPs and P80-CuONPs exhibited the highest SI (range of 2.0–3.0), which is comparable for the SI for doxorubicin, a clinically used chemotherapeutic. The potential mechanism regarding the selective toxicity of PEG-CuONPs and P80-CuONPs in cancer cells may be attributed to the molecular structure of PEG and P80. PEG is a polymer with unique hydrophilicity and electrical neutrality hydroxyl groups. PEG-coated metal nanoparticles showed enhanced cellular internalization by cancer cells. The effects of PEG on selective toxicity to cancer cells have been reported in several studies [79,80], which are in line with our results. P80 is an amphiphilic molecule consisting of a polar head group and a non-polar hydrocarbon tail. Its non-polar tail can interact with the surrounding medium, while the polar head can interact with the metal atoms of CuONPs. This leads that the CuONPs capped with PEG or P80 enhanced biocompatibility for normal living cells. It is further important to note that P80 is a P-glycoprotein inhibitor [81]. Therefore, P80-coated CuONPs likely have the capability to inhibit the P-glycoprotein mediated exocytosis, leading to relatively increased copper oxide concentrations in the cancer cells. In addition, the anticancer activity of CuONPs depends on their particle size, and the smaller the size, the higher the activity. Both PEG and P80 can prevent the aggregation of CuONPs, thereby enhancing their colloidal stability and preventing uncontrolled growth of CuONPs, which likely helps to maintain their anticancer activity.

**Table 3:** IC_50_ values of capping agents against the leukemic cell lines.

Capping agents	IC_50_ values (μg/mL)*		
	KG1a	K562	Molt4
PEG	>100.0^b^	>100.0^b^	>100.0^b^
P80	>100.0^b^	>100.0^b^	>100.0^b^
Tx	9.6 ± 0.4^a^	48.1 ± 8.4^a^	33.7 ± 5.0^a^
SLES	>100.0^b^	>100.0^b^	70.7 ± 2.1^b^
Man	>100.0^b^	>100.0^b^	>100.0^b^
G	>100.0^b^	>100.0^b^	>100.0^b^

* Results are expressed as mean±SD (n = 3). Lowercase letters indicate significantly different (*p* < 0.05).

## Conclusion

This study shows that CuONPs synthesized using the extract from the *C. sappan* plant and different capping agents have specific index values comparable to the clinically used anticancer drug doxorubicin. The SI value is further dependent on the capping agent used. Particularly, the CuONPs prepared using PEG and P80 as capping agents showed the best performance in terms of SI and these particles are therefore interesting systems for further preclinical development.

## Supporting information

S1 File Raw data of our results. (PDF)
