# Supplementary material for: Influence of capping agents on physicochemical properties and leukemic cytotoxicity of copper oxide nanoparticles biosynthesized using Caesalpinia sappan extract
Source: PLoS One. 2025 Jun 26;20(6):e0326791. doi: 10.1371/journal.pone.0326791 (PMC12200837; doi:10.1371/journal.pone.0326791)
Supplement: S1 File — (PDF) [file pone.0326791.s001.pdf]

Raw Data of our Results

S1 Table. Raw data of reducing activity of an extract of *C. Sappan*.

**Sample**	**Reducing activity**				
	**1st experiment**	**2nd experiment**	**3rd experiment**	**Average**	**SD**
The extract of *C. sappan*	57.7	57.7	57.9	57.8	0.1

S2 Table. Raw data of yield of synthesized CuONPs.

**Capping agent**	**Sample**	**yield**				
		**1st experiment**	**2nd experiment**	**3rd experiment**	**Average**	**SD**
-	CuONPs*	69.1	85.9	75.3	75.1	7.7
Gelatin	GCS-CuONPs*	71.7	74.7	78.0	74.8	4.5
PEG400	PEG-CuONPs*	90.5	126.2	85.3	96.2	20.3
Polysorbate80	P80-CuONPs*	88.5	92.7	60.3	78.7	14.5
Octyl phenol ethoxylate	Tx-CuONPs*	70.4	76.3	81.7	76.0	8.0
Sodium lauryl ether sulfate	SLES-CuONPs*	90.9	92.8	96.1	91.8	1.3
Mannitol	Man-CuONPs*	79.6	81.0	82.5	81.8	1.1
Gelatin	G-CuONPs**	56.1	59.4	57.7	57.7	2.3

* prepared with the plant extract, ** prepared with sodium borohydide, *** results are expressed as the mean and standard deviation of three independently prepared batches.

S3 Table. Raw data of particle size of synthesized CuONPs.

Capping agent	Sample	Particle size (nm)				
		1st experiment	2nd experiment	3rd experiment	Average	SD
-	CuONPs*	69.1	85.9	75.3	75.1	7.7
Gelatin	GCS - CuONPs*	71.7	74.7	78.0	74.8	4.5
PEG400	PEG- CuONPs*	90.5	126.2	85.3	96.2	20.3
Polysorbate80	P80- CuONPs*	88.5	92.7	60.3	78.7	14.5
Octyl phenol ethoxylate	Tx- CuONPs*	70.4	76.3	81.7	76.0	8.0
Sodium lauryl ether sulfate	SLES- CuONPs*	90.9	92.8	96.1	91.8	1.3
Mannitol	Man- CuONPs*	79.6	81.0	82.5	81.8	1.1
Gelatin	G- CuONPs**	56.1	59.4	57.7	57.7	2.3

* prepared with the plant extract, ** prepared with sodium borohydide, *** results are expressed as the mean and standard deviation of three independently prepared batches.

S4 Table. Raw data of polydispersity index (PDI) of synthesized CuONPs.

Capping agent	Sample	PDI				
		1st experiment	2nd experiment	3rd experiment	Average	SD
-	CuONPs*	0.280	0.284	0.327	75.1	7.7
Gelatin	GCS - CuONPs*	0.636	0.603	0.844	74.8	4.5
PEG400	PEG-CuONPs*	0.160	0.176	0.183	96.2	20.3
Polysorbate80	P80-CuONPs*	0.370	0.354	0.421	78.7	14.5
Octyl phenol ethoxylate	Tx-CuONPs*	0.313	0.322	0.327	76.0	8.0
Sodium lauryl ether sulfate	SLES-CuONPs*	0.251	0.242	0.323	91.8	1.3
Mannitol	Man-CuONPs*	0.244	0.214	0.245	81.8	1.1
Gelatin	G-CuONPs**	0.352	0.365	0.374	57.7	2.3

* prepared with the plant extract, ** prepared with sodium borohydide, *** results are expressed as the mean and standard deviation of three independently prepared batches.

S5 Table. Raw data of zeta potential of synthesized CuONPs.

Capping agent	Sample	Zeta potential (mV)				
		1st experiment	2nd experiment	3rd experiment	Average	SD
-	CuONPs*	–42.8	–42.4	–42.2	–42.5	0.3
Gelatin	GCS-CuONPs*	–1.9	–1.21	–1.81	–1.81	0.6
PEG400	PEG-CuONPs*	–35.4	–36.7	–34.5	–34.5	1.3
Polysorbate80	P80-CuONPs*	–30.9	–30.1	–29.5	–30.2	0.7
Octyl phenol ethoxylate	Tx-CuONPs*	–31.1	–31.4	–36.2	–32.9	2.9
Sodium lauryl ether sulfate	SLES-CuONPs*	–35.4	–35.1	–33.1	–34.2	0.9
Mannitol	Man-CuONPs*	–38.4	–39.0	–37.2	–38.2	1.1
Gelatin	G-CuONPs**	–2.37	–2.33	–2.71	–2.71	0.2

* prepared with the plant extract, ** prepared with sodium borohydide, *** results are expressed as the mean and standard deviation of three independently prepared batches.

Cytotoxicity analysis of CuONPs

S6 Table. Raw data of IC_{50} values of *C. sappan* extract, copper sulfate, and the synthesized CuONPs against PBMC.

Samples	IC50 values				
	1st volunteer	2nd volunteer	3rd volunteer	Average	SD
C. sappan extract	60.0	66.2	74.0	66.7	4.0
Copper sulfate	77.5	71.7	72.5	73.9	1.8
CuONPs	43.8	53.3	65.4	54.1	6.2
PEG-CuONPs	60.9	77.4	79.1	72.5	5.8
P80-CuONPs	89.9	86.0	79.2	85.0	3.1
Tx-CuONPs	25.0	32.0	24.9	27.3	2.3
SLES-CuONPs	28.4	36.6	37.4	34.1	2.9
Man-CuONPs	92.9	88.3	80.9	87.4	3.5
G-CuONPs	70.5	57.6	59.9	62.6	4.0
Doxorubicin	1.8	1.8	1.8	1.8	0.0

S7 Table. Raw data of IC_{50} values of *C. sappan* extract, copper sulfate, and the synthesized CuONPs against KG1a.

Samples	IC50 values				
	1st experiment	2nd experiment	3rd experiment	Average	SD
C. sappan extract	23.8	22.2	16.7	20.9	3.7
Copper sulfate	>100.0	>100.0	>100.0	>100.0	
CuONPs	82.2	86.7	69.5	79.5	9.0
PEG-CuONPs	23.2	35.6	29.0	29.3	6.2
P80-CuONPs	29.7	27.3	27.6	28.2	1.3
Tx-CuONPs	56.9	56.4	57.9	57.1	0.8
SLES-CuONPs	36.0	35.6	35.3	35.6	3.4
Man-CuONPs	>100.0	>100.0	>100.0	>100.0	
G-CuONPs	>100.0	>100.0	>100.0	>100.0	
Doxorubicin	0.7	0.7	0.6	0.7	0.1

S8 Table. **Raw data of IC_{50} values of *C. sappan* extract, copper sulfate, and the synthesized CuONPs against K562**.

Samples	IC_{50} values				
	1st experiment	2nd experiment	3rd experiment	Average	SD
C. sappan extract	>100.0	>100.0	>100.0	>100.0	
Copper sulfate	>100.0	>100.0	>100.0	>100.0	
CuONPs	69.9	74.8	68.5	73.3	4.2
PEG-CuONPs	24.7	23.3	31.0	26.3	4.1
P80-CuONPs	31.3	33.1	42.7	35.7	6.1
Tx-CuONPs	22.3	25.7	28.5	25.5	2.5
SLES-CuONPs	27.7	32.9	30.8	30.5	2.6
Man-CuONPs	46.6	59.6	58.1	54.7	7.1
G-CuONPs	84.0	83.7	83.9	83.9	0.1
Doxorubicin	0.5	0.4	0.5	0.5	0.1

S9 Table. **Raw data of IC_{50} values of *C. sappan* extract, copper sulfate, and the synthesized CuONPs against Molt4**.

Samples	IC_{50} values				
	1st experiment	2nd experiment	3rd experiment	Average	SD
C. sappan extract	>100.0	>100.0	>100.0	>100.0	
Copper sulfate	>100.0	>100.0	>100.0	>100.0	
CuONPs	97.4	91.9	83.1	90.8	7.2
PEG-CuONPs	30.1	32.6	25.0	29.3	3.9
P80-CuONPs	45.1	41.9	38.7	41.9	3.2
Tx-CuONPs	84.4	92.3	94.7	90.5	5.4
SLES-CuONPs	78.3	73.8	68.9	73.7	4.7
Man-CuONPs	>100.0	>100.0	>100.0	>100.0	
G-CuONPs	>100.0	>100.0	>100.0	>100.0	
Doxorubicin	0.7	0.7	0.6	0.7	0.1

S10 Table. Raw data of cytotoxicity of *C. sappan* extract against PBMC.

C. sappan extract	Cell viability (%)				
	1st volunteer	2nd volunteer	3rd volunteer	Average	SD
0.0	100.0	100.0	100.0	100.0	0.0
3.1	96.8	98.3	96.3	97.1	1.0
6.3	88.1	95.0	85.5	89.5	4.9
12.5	82.9	86.6	83.7	84.4	1.9
25.0	80.2	70.7	81.6	77.5	5.9
50.0	59.4	58.1	60.6	59.4	1.3
100.0	27.9	39.7	38.4	35.3	6.5

S11 Table Raw data of cytotoxicity of copper sulfate against PBMC.

Copper sulfate	Cell viability (%)				
	1st volunteer	2nd volunteer	3rd volunteer	Average	SD
0.0	100.0	100.0	100.0	100.0	0.0
12.5	89.4	80.8	78.8	83.0	5.6
25.0	84.4	76.7	63.2	74.8	10.7
50.0	71.2	62.8	55.2	63.1	8.0
100.0	32.7	33.2	43.6	36.5	6.2
200.0	10.7	11.0	32.7	18.2	12.6
400.0	7.5	7.6	11.1	8.7	2.0

S12 Table. Raw data of cytotoxicity of CuONPs extract PBMC.

CuONPs	Cell viability (%)				
	1st volunteer	2nd volunteer	3rd volunteer	Average	SD
0.0	100.0	100.0	100.0	100.0	0.0
3.1	87.5	99.2	95.8	94.2	6.0
6.3	79.4	96.2	89.4	88.4	8.5
12.5	74.0	83.8	71.8	76.5	6.4
25.0	64.0	81.9	71.5	72.5	9.0
50.0	45.4	51.9	59.5	52.2	7.1
100.0	36.3	23.1	28.7	29.3	6.6

S13 Table. Raw data of cytotoxicity of PEG-CuONPs against PBMC.

PEG-CuONPs	Cell viability (%)				
	1st volunteer	2nd volunteer	3rd volunteer	Average	SD
0.0	100.0	100.0	100.0	100.0	0.0
3.1	95.8	92.0	91.1	92.9	2.5
6.3	88.1	98.8	87.3	91.4	6.4
12.5	82.5	90.7	76.5	83.3	7.1
25.0	65.6	80.9	77.7	74.7	8.1
50.0	52.8	66.6	67.2	62.2	8.1
100.0	40.0	36.3	37.6	38.0	1.9

S14 Table. Raw data of cytotoxicity of P80-CuONPs against PBMC.

P80-CuONPs	Cell viability (%)				
	1st volunteer	2nd volunteer	3rd volunteer	Average	SD
0.0	100.0	100.0	100.0	100.0	0.0
3.1	90.4	102.6	101.2	98.1	6.7
6.3	94.1	98.0	90.2	94.1	3.9
12.5	93.4	100.3	86.6	93.4	6.8
25.0	90.1	100.1	80.1	90.1	10.0
50.0	58.5	76.2	71.5	68.7	9.2
100.0	47.8	39.8	34.6	40.8	6.7

S15 Table. Raw data of cytotoxicity of Tx-CuONPs against PBMC.

Tx-CuONPs	Cell viability (%)				
	1st volunteer	2nd volunteer	3rd volunteer	Average	SD
0.0	100.0	100.0	100.0	100.0	0.0
3.1	87.6	94.6	80.7	87.6	6.9
6.3	82.6	89.8	75.5	82.6	7.1
12.5	84.8	94.8	74.8	84.8	10.0
25.0	50.1	59.2	49.9	53.0	5.3
50.0	36.7	26.3	30.5	31.2	5.2
100.0	23.0	12.0	12.6	15.9	6.2

S16 Table. Raw data of cytotoxicity of SLES-CuONPs against PBMC.

SLES-CuONPs	Cell viability (%)				
	1st volunteer	2nd volunteer	3rd volunteer	Average	SD
0.0	100.0	100.0	100.0	100.0	0.0
3.1	81.4	95.3	85.6	87.5	7.1
6.3	87.4	92.9	82.0	87.4	5.5
12.5	83.0	90.9	75.1	83.0	7.9
25.0	50.0	55.6	66.9	57.5	8.6
50.0	25.5	43.5	32.7	33.9	9.0
100.0	19.2	20.7	14.5	18.2	3.2

S17 Table. Raw data of cytotoxicity of Man-CuONPs against PBMC.

Man-CuONPs	Cell viability (%)				
	1st volunteer	2nd volunteer	3rd volunteer	Average	SD
0.0	100.0	100.0	100.0	100.0	0.0
12.5	92.3	97.1	81.5	90.3	8.0
25.0	75.2	92.6	79.9	82.6	9.0
50.0	58.3	78.8	72.1	69.7	10.5
100.0	48.6	41.2	47.3	45.7	4.0
200.0	19.3	17.1	33.3	23.3	8.8
400.0	13.5	11.6	12.2	12.4	1.0

S18 Table. Raw data of cytotoxicity of G-CuONPs against PBMC.

G-CuONPs	Cell viability (%)				
	1st volunteer	2nd volunteer	3rd volunteer	Average	SD
0.0	100.0	100.0	100.0	100.0	0.0
12.5	89.7	97.6	88.0	91.8	5.1
25.0	79.1	64.4	85.5	76.3	10.8
50.0	71.0	64.1	78.7	71.3	7.3
100.0	60.2	51.7	57.2	56.4	4.3
200.0	35.3	40.6	20.8	32.2	10.2
400.0	12.1	18.0	9.0	13.0	4.6

S19 Table. Raw data of cytotoxicity of doxorubicin against PBMC.

Doxorubicin	Cell viability (%)				
	1st volunteer	2nd volunteer	3rd volunteer	Average	SD
0.0	100.0	100.0	100.0	100.0	0.0
0.031	86.9	88.5	98.9	93.7	7.3
0.063	91.5	89.5	98.7	94.1	6.5
0.125	87.4	86.7	92.6	89.6	4.1
0.250	72.6	78.4	88.8	83.6	7.4
0.500	79.4	79.7	76.8	78.2	2.1
1.000	59.5	58.6	54.3	56.5	3.0
2.000	23.8	23.4	27.2	25.3	2.6

KG1a

S20 Table. Raw data of cytotoxicity of *C. sappan* extract against KG1a.

C. sappan extract	Cell viability (%)				
	1st experiment	2nd experiment	3rd experiment	Average	SD
0.0	100.0	100.0	100.0	100.0	0.0
6.3	86.3	88.2	91.3	88.6	2.5
12.5	69.8	69.4	58.7	66.0	6.3
25.0	47.9	44.4	33.0	41.7	7.8
50.0	22.6	20.2	26.9	23.3	3.4
100.0	19.8	24.3	21.3	21.8	2.3

S21 Table. Raw data of cytotoxicity of copper sulfate against KG1a.

Copper sulfate	Cell viability (%)				
	1st experiment	2nd experiment	3rd experiment	Average	SD
0.0	100.0	100.0	100.0	100.0	0.0
12.5	82.7	84.4	89.0	85.4	3.3
25.0	80.9	79.3	88.4	82.9	4.9
50.0	70.7	70.8	79.0	73.5	4.8
100.0	61.1	60.1	64.6	61.9	2.4
200.0	41.2	46.5	43.4	43.7	2.6
400.0	13.2	15.2	12.9	13.8	1.3

S22 Table. Raw data of cytotoxicity of CuONPs against KG1a.

CuONPs	Cell viability (%)				
	1st experiment	2nd experiment	3rd experiment	Average	SD
0.0	100.0	100.0	100.0	100.0	0.0
3.1	81.9	88.5	100.0	90.1	9.2
6.3	79.4	88.3	94.3	87.3	7.5
12.5	72.6	84.0	80.4	79.0	5.9
25.0	67.5	74.6	81.8	74.6	7.2
50.0	55.9	69.5	63.9	63.1	6.8
100.0	46.8	43.0	28.1	39.3	9.8

S23 Table. Raw data of cytotoxicity of PEG-CuONPs against KG1a.

PEG-CuONPs	Cell viability (%)				
	1st experiment	2nd experiment	3rd experiment	Average	SD
0.0	100.0	100.0	100.0	100.0	0.0
3.1	87.7	82.1	83.9	84.6	2.9
6.3	76.4	70.5	77.3	74.7	3.7
12.5	66.6	65.3	68.0	66.6	1.4
25.0	47.2	59.6	53.7	53.5	6.2
50.0	28.9	37.0	30.5	32.1	4.3
100.0	7.4	8.5	10.1	8.7	1.3

S24 Table. Raw data of cytotoxicity of P80-CuONPs against KG1a.

P80-CuONPs	Cell viability (%)				
	1st experiment	2nd experiment	3rd experiment	Average	SD
0.0	100.0	100.0	100.0	100.0	0.0
3.1	87.1	88.8	85.3	87.1	2.5
6.3	74.6	78.1	73.5	75.4	2.4
12.5	70.5	69.7	65.7	68.7	2.6
25.0	57.4	52.7	54.8	55.0	2.4
50.0	18.2	23.6	9.3	17.1	7.2
100.0	8.1	7.9	8.0	8.0	0.1

S25 Table. Raw data of cytotoxicity of Tx-CuONPs against KG1a.

Tx-CuONPs	Cell viability (%)				
	1st experiment	2nd experiment	3rd experiment	Average	SD
0.0	100.0	100.0	100.0	100.0	0.0
3.1	85.2	81.7	79.6	82.2	2.8
6.3	83.6	88.5	80.6	84.2	4.0
12.5	78.3	83.0	69.9	77.1	6.6
25.0	64.4	67.9	65.9	66.1	1.7
50.0	52.8	53.0	54.5	53.5	0.9
100.0	32.2	29.4	26.0	29.2	3.1

S26 Table. Raw data of cytotoxicity of SLES-CuONPs against KG1a.

SLES-CuONPs	Cell viability (%)				
	1st experiment	2nd experiment	3rd experiment	Average	SD
0.0	100.0	100.0	100.0	100.0	0.0
6.3	85.2	76.8	70.0	78.1	6.6
12.5	79.6	68.0	71.0	71.0	7.6
25.0	61.3	62.9	58.2	60.4	3.1
50.0	35.7	32.4	38.2	29.7	7.7
100.0	9.6	7.5	12.1	8.3	1.1
200.0	7.1	8.2	8.9	7.7	0.6

S27 Table. Raw data of cytotoxicity of Man-CuONPs against KG1a.

Man-CuONPs	Cell viability (%)				
	1st experiment	2nd experiment	3rd experiment	Average	SD
0.0	100.0	100.0	100.0	100.0	0.0
12.5	86.7	86.7	89.6	87.6	1.7
25.0	72.1	75.7	87.5	78.4	8.1
50.0	64.7	69.3	77.3	70.4	6.4
100.0	58.7	53.8	58.8	57.1	2.9
200.0	15.2	25.7	27.5	22.8	6.7
400.0	7.7	7.6	6.7	7.3	0.5

S28 Table. Raw data of cytotoxicity of G-CuONPs against KG1a.

G-CuONPs	Cell viability (%)				
	1st experiment	2nd experiment	3rd experiment	Average	SD
0.0	100.0	100.0	100.0	100.0	0.0
6.3	85.3	93.7	90.3	89.5	6.0
12.5	82.5	92.7	92.0	89.1	5.7
25.0	74.7	88.5	87.2	83.5	7.7
50.0	76.8	78.2	80.5	78.5	1.9
100.0	66.3	60.8	66.1	64.4	3.1
200.0	37.1	34.5	30.2	33.9	3.5

S29 Table. Raw data of cytotoxicity of doxorubicin against KG1a.

Doxorubicin	Cell viability (%)				
	1st experiment	2nd experiment	3rd experiment	Average	SD
0.000	100.0	100.0	100.0	100.0	0.0
0.002	92.8	88.6	99.8	93.7	5.6
0.004	100.4	95.9	98.3	98.2	2.3
0.008	97.3	92.9	98.3	96.2	2.9
0.016	102.3	97.7	95.1	98.4	3.7
0.031	105.5	100.8	93.7	100.0	6.0
0.063	93.6	97.9	86.4	92.6	5.8
0.125	97.0	92.7	94.0	94.8	3.1
0.250	80.9	77.3	74.9	77.7	3.0
0.500	67.0	64.0	54.8	62.0	6.4
1.000	32.5	31.0	34.6	32.7	1.8

K562

S30 Table. Raw data of cytotoxicity of *C. sappan* extract against K562.

C. sappan extract	Cell viability (%)				
	1st experiment	2nd experiment	3rd experiment	Average	SD
0.0	100.0	100.0	100.0	100.0	0.0
3.1	91.7	98.9	97.3	95.9	3.8
6.3	93.0	97.1	94.5	94.9	2.0
12.5	95.9	92.9	94.8	94.5	1.5
25.0	100.7	92.2	95.7	96.2	4.3
50.0	108.1	103.5	100.3	104.0	3.9
100.0	102.9	96.0	111.8	103.6	7.9

S31 Table. Raw data of cytotoxicity of copper sulfate against K562.

Copper sulfate	Cell viability (%)				
	1st experiment	2nd experiment	3rd experiment	Average	SD
0.0	100.0	100.0	100.0	100.0	0.0
12.5	77.8	81.6	88.2	82.5	5.3
25.0	69.8	78.2	88.2	78.8	9.2
50.0	68.1	85.0	80.3	77.8	8.8
100.0	59.1	51.5	54.7	55.1	3.8
200.0	32.0	42.6	31.8	35.4	6.2
400.0	10.9	7.9	6.6	8.5	2.2

S32 Table. Raw data of cytotoxicity of CuONPs against K562.

CuONPs	Cell viability (%)				
	1st experiment	2nd experiment	3rd experiment	Average	SD
0.0	100.0	100.0	100.0	100.0	0.0
3.1	82.0	80.5	78.7	80.4	1.7
6.3	84.7	89.3	71.1	81.7	9.5
12.5	80.7	87.2	69.8	79.2	8.8
25.0	70.4	77.4	63.3	70.4	7.0
50.0	68.2	76.9	59.5	68.2	8.7
100.0	22.5	22.6	33.7	26.3	6.5

S33 Table. Raw data of cytotoxicity of PEG-CuONPs against K562.

PEG-CuONPs	Cell viability (%)				
	1st experiment	2nd experiment	3rd experiment	Average	SD
0.0	100.0	100.0	100.0	100.0	0.0
3.1	90.2	94.0	93.0	92.4	2.0
6.3	96.1	91.3	89.3	92.2	3.5
12.5	82.2	88.3	88.8	86.4	3.7
25.0	49.3	44.0	56.7	50.0	6.4
50.0	15.7	21.1	28.7	21.8	6.5
100.0	7.9	8.9	9.2	8.7	0.7

S34 Table. Raw data of cytotoxicity of P80-CuONPs against K562.

P80-CuONPs	Cell viability (%)				
	1st experiment	2nd experiment	3rd experiment	Average	SD
0.0	100.0	100.0	100.0	100.0	0.0
3.1	93.3	93.2	91.4	92.7	1.1
6.3	88.5	84.8	88.6	87.3	2.2
12.5	84.9	78.3	89.4	84.2	5.6
25.0	58.2	56.1	61.1	58.5	2.5
50.0	25.7	37.3	45.4	36.1	9.9
100.0	8.2	8.0	12.8	9.7	2.7

S35 Table. Raw data of cytotoxicity of Tx-CuONPs against K562.

Tx-CuONPs	Cell viability (%)				
	1st experiment	2nd experiment	3rd experiment	Average	SD
0.0	100.0	100.0	100.0	100.0	0.0
3.1	86.2	87.3	86.0	86.5	0.7
6.3	89.9	79.4	82.9	84.1	5.3
12.5	62.8	69.7	69.4	67.3	3.9
25.0	46.4	50.8	53.5	50.2	3.6
50.0	10.5	22.6	28.5	20.5	9.2
100.0	7.0	9.1	7.4	7.8	1.1

S36Table. Raw data of cytotoxicity of SLES-CuONPs against K562.

SLES-CuONPs	Cell viability (%)				
	1st experiment	2nd experiment	3rd experiment	Average	SD
0.0	100.0	100.0	100.0	100.0	0.0
3.1	92.9	88.6	95.5	92.4	3.5
6.3	85.9	88.9	90.7	88.5	2.4
12.5	83.7	79.2	77.6	80.2	3.2
25.0	53.5	59.3	56.4	56.4	2.9
50.0	21.6	29.9	28.9	26.8	4.5
100.0	8.2	9.6	7.7	8.5	1.0

S37 Table. Raw data of cytotoxicity of Man-CuONPs against K562.

Man-CuONPs	Cell viability (%)				
	1st experiment	2nd experiment	3rd experiment	Average	SD
0.0	100.0	100.0	100.0	100.0	0.0
12.5	87.4	89.1	97.3	91.2	5.3
25.0	76.7	84.2	96.7	85.9	10.1
50.0	45.7	57.4	53.4	52.2	5.9
100.0	22.4	18.9	32.2	24.5	6.9
200.0	15.6	7.0	8.7	10.4	4.5
400.0	4.8	6.1	6.7	5.9	1.0

S38 Table. Raw data of cytotoxicity of G-CuONPs against K562.

G-CuONPs	Cell viability (%)				
	1st experiment	2nd experiment	3rd experiment	Average	SD
0.0	100.0	100.0	100.0	100.0	0.0
6.3	96.2	93.6	90.7	93.5	2.7
12.5	93.5	87.8	78.1	90.6	4.0
25.0	89.8	89.9	76.2	89.8	0.1
50.0	62.8	69.9	67.6	66.3	5.0
100.0	44.0	40.4	67.2	42.2	2.5
200.0	5.1	6.5	19.8	5.8	1.0

S39 Table. Raw data of cytotoxicity of doxorubicin against K562.

Doxorubicin	Cell viability (%)				
	1st experiment	2nd experiment	3rd experiment	Average	SD
0.000	100.0	100.0	100.0	100.0	0.0
0.002	90.1	98.4	100.0	95.1	7.0
0.004	87.6	95.8	104.0	95.8	8.2
0.008	88.9	94.6	102.3	95.3	6.8
0.016	85.0	81.6	98.0	88.2	8.6
0.031	81.8	75.1	89.2	82.1	7.1
0.063	72.1	63.6	83.9	73.2	10.2
0.125	72.2	68.0	72.0	70.7	2.4
0.250	64.4	66.1	67.9	66.1	1.7
0.500	48.9	45.3	52.5	48.9	3.6
1.000	27.4	28.5	26.3	27.4	1.1

Molt4

S40 Table. Raw data of cytotoxicity of *C. sappan* extract against Molt4.

C. sappan extract	Cell viability (%)				
	1st experiment	2nd experiment	3rd experiment	Average	SD
0.0	0.0	100.0	100.0	100.0	100.0
6.3	3.1	100.0	102.4	103.6	102.0
12.5	6.3	101.0	105.1	103.7	103.3
25.0	12.5	100.6	103.4	104.2	102.7
50.0	25.0	100.9	103.5	104.9	103.1
100.0	50.0	110.3	108.1	108.0	108.8
200.0	100.0	108.2	114.1	115.8	112.7

S41 Table. Raw data of cytotoxicity of copper sulfate against Molt4.

Copper sulfate	Cell viability (%)				
	1st experiment	2nd experiment	3rd experiment	Average	SD
0.0	100.0	100.0	100.0	100.0	0.0
12.5	97.9	99.2	98.5	98.5	0.6
25.0	92.5	88.7	90.6	90.6	1.9
50.0	77.3	79.9	73.5	76.9	3.3
100.0	70.0	67.2	69.5	68.9	1.5
200.0	51.0	52.8	52.0	52.0	0.9
400.0	13.4	14.1	16.1	14.5	1.4

S42 Table. Raw data of cytotoxicity of CuONPs against Molt4.

CuONPs	Cell viability (%)				
	1st experiment	2nd experiment	3rd experiment	Average	SD
0.0	100.0	100.0	100.0	100.0	0.0
3.1	96.1	87.0	96.0	93.1	5.2
6.3	92.1	92.0	93.6	92.6	0.9
12.5	95.0	94.0	88.8	92.6	3.4
25.0	74.8	78.4	79.2	77.5	2.3
50.0	63.8	76.6	74.7	71.7	6.9
100.0	49.2	44.9	37.4	43.8	6.0

S43 Table. Raw data of cytotoxicity of PEG-CuONPs against Molt4.

PEG-CuONPs	Cell viability (%)				
	1st experiment	2nd experiment	3rd experiment	Average	SD
0.0	100.0	100.0	100.0	100.0	0.0
3.1	103.9	103.9	101.0	102.9	1.7
6.3	91.0	102.6	91.6	95.1	6.5
12.5	81.6	98.0	86.6	88.7	8.4
25.0	60.2	68.1	50.1	59.5	9.1
50.0	10.2	8.6	12.8	10.5	2.1
100.0	4.8	5.0	5.6	5.1	0.4

S44 Table. Raw data of cytotoxicity of P80-CuONPs against Molt4.

P80-CuONPs	Cell viability (%)				
	1st experiment	2nd experiment	3rd experiment	Average	SD
0.0	100.0	100.0	100.0	100.0	0.0
3.1	91.6	89.5	91.5	90.9	1.2
6.3	87.4	98.3	89.0	91.6	5.9
12.5	83.1	96.0	81.6	86.9	7.9
25.0	74.7	88.2	72.3	78.4	8.6
50.0	43.9	31.6	31.5	35.7	7.1
100.0	22.0	22.0	6.0	16.7	9.2

S45 Table. Raw data of cytotoxicity of Tx-CuONPs against Molt4.

P80-CuONPs	Cell viability (%)				
	1st experiment	2nd experiment	3rd experiment	Average	SD
0.0	100.0	100.0	100.0	100.0	0.0
3.1	92.3	93.7	93.1	93.0	0.7
6.3	91.9	88.3	90.0	90.1	1.8
12.5	88.8	87.3	89.1	88.4	1.0
25.0	78.5	81.0	80.4	80.0	1.3
50.0	69.5	70.0	69.5	69.7	0.3
100.0	41.2	46.4	47.7	45.1	3.5

S46 Table. Raw data of cytotoxicity of SLES-CuONPs against Molt4.

SLES-CuONPs	Cell viability (%)				
	1st experiment	2nd experiment	3rd experiment	Average	SD
0.0	100.0	100.0	100.0	100.0	0.0
3.1	91.0	89.2	97.3	92.5	4.3
6.3	102.4	86.8	103.5	97.6	9.4
12.5	107.2	87.8	100.0	98.3	9.8
25.0	101.7	81.4	90.5	91.2	10.2
50.0	66.9	70.1	64.6	67.2	2.8
100.0	37.1	27.9	26.0	30.3	5.9

S47 Table. Raw data of cytotoxicity of Man-CuONPs against Molt4.

Man-CuONPs	Cell viability (%)				
	1st experiment	2nd experiment	3rd experiment	Average	SD
0.0	100.0	100.0	100.0	100.0	0.0
12.5	96.5	92.3	94.7	94.5	2.1
25.0	98.1	94.2	93.1	95.1	2.6
50.0	97.7	80.3	86.6	88.2	8.8
100.0	87.9	74.3	82.9	81.7	6.8
200.0	64.5	65.1	59.6	63.1	3.0
400.0	14.5	7.5	17.0	13.0	4.9

S48 Table. Raw data of cytotoxicity of G-CuONPs against Molt4.

G-CuONPs	Cell viability (%)				
	1st experiment	2nd experiment	3rd experiment	Average	SD
0.0	100.0	100.0	100.0	100.0	0.0
6.3	105.8	89.3	98.0	97.7	8.2
12.5	99.4	91.2	98.6	96.4	4.5
25.0	92.8	94.1	79.8	88.9	7.9
50.0	73.0	79.3	69.9	74.0	4.8
100.0	50.2	52.6	51.4	51.4	1.2
200.0	7.5	11.3	6.2	8.3	2.7

S49 Table. Raw data of cytotoxicity of G-CuONPs against Molt4.

G-CuONPs	Cell viability (%)				
	1st experiment	2nd experiment	3rd experiment	Average	SD
0.000	100.0	100.0	100.0	100.0	0.0
0.008	94.7	94.8	95.7	95.1	0.5
0.016	91.7	92.8	93.3	92.6	0.8
0.031	94.5	100.9	89.3	94.9	5.8
0.063	100.5	112.1	97.3	103.3	7.8
0.125	118.8	102.0	113.0	111.3	8.5
0.250	78.3	79.8	66.8	74.9	7.1
0.500	48.1	55.1	46.7	50.0	4.5
1.000	33.4	37.4	48.1	39.6	7.6

Cytotoxicity analysis of capping agents

S50 Table. Raw data of IC_{50} values of capping agents against KG1a.

Samples	IC_{50} values				
	1st experiment	2nd experiment	3rd experiment	Average	SD
PEG	>100.0	>100.0	>100.0	>100.0	4.0
P80	>100.0	>100.0	>100.0	>100.0	1.8
Tx	9.2	10.0	9.6	9.6	0.4
SLES	>100.0	>100.0	>100.0	>100.0	5.8
Man	>100.0	>100.0	>100.0	>100.0	3.1
G	>100.0	>100.0	>100.0	>100.0	2.3

S51 Table. Raw data of IC_{50} values of capping agents against K562.

Samples	IC_{50} values				
	1st experiment	2nd experiment	3rd experiment	Average	SD
PEG	>100.0	>100.0	>100.0	>100.0	4.0
P80	>100.0	>100.0	>100.0	>100.0	1.8
Tx	38.6	54.3	51.7	48.2	8.4
SLES	>100.0	>100.0	>100.0	>100.0	5.8
Man	>100.0	>100.0	>100.0	>100.0	3.1
G	>100.0	>100.0	>100.0	>100.0	2.3

S52 Table. Raw data of IC_{50} values of capping agents against Molt4.

Samples	IC_{50} values				
	1st experiment	2nd experiment	3rd experiment	Average	SD
PEG	>100.0	>100.0	>100.0	>100.0	4.0
P80	>100.0	>100.0	>100.0	>100.0	1.8
Tx	39.4	31.6	30.1	33.7	5.0
SLES	>100.0	>100.0	>100.0	>100.0	5.8
Man	>100.0	>100.0	>100.0	>100.0	3.1
G	>100.0	>100.0	>100.0	>100.0	2.3

S53 Table. Raw data of cytotoxicity of Tx against KG1a.

Tx	Cell viability (%)				
	1st experiment	2nd experiment	3rd experiment	Average	SD
0.0	100.0	100.0	100.0	100.0	0.0
3.1	74.1	64.4	73.0	70.5	5.3
6.3	61.9	58.9	63.6	61.5	2.4
12.5	37.0	44.0	37.9	39.6	3.8
25.0	28.4	31.4	26.8	28.8	2.3
50.0	20.7	18.2	19.6	19.5	1.2
100.0	6.7	8.4	6.3	7.1	1.1

S54 Table. Raw data of cytotoxicity of Tx against K562.

Tx	Cell viability (%)				
	1st experiment	2nd experiment	3rd experiment	Average	SD
0.0	100.0	100.0	100.0	100.0	0.0
3.1	80.9	88.3	81.6	83.6	4.1
6.3	76.2	66.5	74.8	72.5	5.2
12.5	72.7	69.0	66.0	69.2	3.3
25.0	64.2	63.2	55.4	60.9	4.8
50.0	38.0	54.1	51.5	47.9	8.6
100.0	5.1	5.5	6.9	5.9	0.9

S55 Table. Raw data of cytotoxicity of Tx against Molt4.

Tx	Cell viability (%)				
	1st experiment	2nd experiment	3rd experiment	Average	SD
0.0	100.0	100.0	100.0	100.0	0.0
3.1	87.0	74.4	82.9	81.5	6.4
6.3	70.6	75.9	65.3	70.6	5.3
12.5	75.5	72.0	65.6	71.0	5.0
25.0	68.2	55.9	58.4	60.8	6.5
50.0	36.5	33.7	17.5	29.2	10.3
100.0	5.1	5.2	5.0	5.1	0.1

S56 table FTIR spectrum of synthesized CuONPs and capping agents

Wave number (cm^{-1})	Bond	Functional groups
CuONPs		
3383	O-H stretch	Hydroxyl groups
1575	C=O stretch	C=O stretching of the carboxylic groups
505	Cu-O stretch	Copper oxide and copper hydroxide
PEG-CuONPs		
3367	O-H stretch	Hydroxyl groups
2863	CH_2 stretch	Alkanes
1347	C-O stretch	Carboxylic acid
500	Cu-O stretch	Copper oxide and copper hydroxide
P80-CuONPs		
2919 and 2846	CH_2 stretch	Alkanes
1731	C=O bond	Carbonyl groups
1564	C-H bending	Alkanes
1097	C-O stretch	Carbonyl groups
500	Cu-O stretch	Copper oxide and copper hydroxide
Tx-CuONPs		
1570	C-H bending	Alkanes
505	Cu-O stretch	Copper oxide and copper hydroxide
SLES-CuONPs		
2919, 2852	CH_2 stretch	Alkanes
1219	SO_2 stretch	Sulfur dioxide
510	Cu-O stretch	Copper oxide and copper hydroxide
Man-CuONPs		
3280	O-H stretch	Hydroxyl groups
2856	CH_2 stretch	Alkanes
615	Cu-O stretch	Copper oxide and copper hydroxide
G-CuONPs		
3428	O-H stretch	Hydroxyl groups
1554	C-N stretch	Amides
626	Cu-O stretch	Copper oxide and copper hydroxide
PEG		
3446	O-H stretch	Hydroxyl groups
2870	CH_2 stretch	Alkanes
1450	C=O stretch	C=O stretching of the carboxylic groups

**Wave number (cm^{-1})**	**Bond**	**Functional groups**
P80		
2872	CH_2 stretch	Alkanes
1730	C=O bond	Carbonyl groups
Tx		
1570	C-H bending	Alkanes
SLES		
2848	CH_2 stretch	Alkanes
1253	SO_2 stretch	Sulfur dioxide
Man		
3372	O-H stretch	Hydroxyl groups
2977	CH_2 stretch	Alkanes
G		
3369	O-H stretch	Hydroxyl groups
1604	C-N stretch	Amides
